# Effect of electrophysiological mapping on non-transmural annulus ablation and atrial fibrillation recurrence prediction after 6 months of Cox-Maze IV procedure

**DOI:** 10.3389/fcvm.2022.931845

**Published:** 2022-07-15

**Authors:** Zhishan Sun, Chengming Fan, Long Song, Hao Zhang, Zenan Jiang, Haoyu Tan, Yaqin Sun, Liming Liu

**Affiliations:** Department of Cardiovascular Surgery, The Second Xiangya Hospital, Central South University, Changsha, China

**Keywords:** Cox-Maze IV, ablation lines, surgery, electrical isolation, mapping, recurrence

## Abstract

**Objective:**

The objective of this study was to observe the safety and efficacy of electrophysiological mapping following the Cox-Maze IV procedure and to investigate whether a correlation exists between recurrence of atrial fibrillation (AF) with the completeness of bidirectional electrical isolation and the inducibility of AF immediately after the Cox-Maze IV procedure.

**Methods:**

Totally, 80 consecutive patients who suffered from aortic valve or mitral valve disease and persistent AF were randomly enrolled into the control group and electrophysiological mapping following the Cox-Maze IV group (Electrophysio-Maze group). In the Electrophysio-Maze group, patients underwent concomitant Cox-Maze procedure and following electrophysiological mapping of ablation lines in mitral isthmus, left atrial “box,” and tricuspid annulus. If the bidirectional electrical isolation of tricuspid annulus ablation line is incomplete, whether to implement supplementary ablation will be independently decided by the operator. Before and after the Cox-Maze IV procedure, AF induction was performed. All patients in both groups were continuously followed-up and underwent electrocardiogram Holter monitoring after 6 months.

**Results:**

In total, 42 Electrophysio-Maze patients and 38 controls were enrolled. Compared with patients in the control group, there were shorter hospital stay, better cardiac remodeling changes, and higher relief from AF during the follow-up period of 6 months in the Electrophysio-Maze group. Within the Electrophysio-Maze group, the rate of incomplete the bidirectional electrical isolation of “box” ablation lines was zero, and the rate of incomplete bidirectional electrical isolation of mitral isthmus ablation line or tricuspid annulus ablation line was 23.8%. After two cases of successful complementary ablation on the tricuspid annulus ablation line, the final incomplete bidirectional electrical isolation of annulus ablation lines was 19.0%. There were correlations between late AF recurrence after 6 months with incomplete bidirectional electrical isolation of annulus ablation lines and AF induction immediately after the Cox-Maze IV procedure.

**Conclusion:**

Electrophysiological mapping following the Cox-Maze procedure is safe and effective. Electrophysiological mapping in the Cox-Maze procedure can find out the non-transmural annulus ablation lines by assessing the completeness of bidirectional electrical isolation of ablation lines, guide supplementary ablation, and predict AF recurrence after 6 months.

## Introduction

Atrial fibrillation (AF) is the most common progressive arrhythmia in the world, which is the main cause of increased risk of stroke, heart failure, peripheral vascular embolism, and other diseases worldwide (1–4). Especially, in patients undergoing mitral valve surgery, the incidence of AF can be as high as 50–80% (5, 6). For this kind of drug-refractory AF, surgery or percutaneous ablation is recommended (7). The first attempt at surgical ablation for AF can be traced back to the 1980s, including left atrial isolation and corridor surgery. However, the success of surgical ablation was not reliably achieved until the development of the Cox-maze surgery in 1987, which then became the gold standard for surgical treatment of AF (8). Genev et al. (9) conducted a comparative study of catheter ablation, hybrid AF ablation, and open maze surgery in 140 patients and found that the sinus cardioversion rates of the three groups were 20.3, 57.9, and 72.7%, respectively, indicating that open maze surgery is significantly better than catheter ablation and hybrid AF ablation. As far as the effect in sinus cardioversion and stroke reduction is concerned, the maze procedure is definitely superior to drug therapy, especially in patients with persistent or long-standing persistent AF (10). However, maze surgery involves complex incisions, which can cause great trauma to the patient’s heart (11). Due to the complexity of the operation, prolonged surgery time, significant trauma, and obvious impact on cardiac function, the classic Cox-maze III surgery is not currently widely used (12). As the maze-III procedure increases risk during surgery, its use is limited (13). To reduce the trauma associated with maze-III, the Cox-Maze IV procedure was introduced in 2002, which is no less effective than maze-III in reducing stroke. Previous studies have confirmed the excellent effect in lowering postoperative stroke of the maze-III procedure (14), yet Murashita and his colleagues found that the incidence of stroke after Cox-Maze IV surgery was no more than that of maze-III procedure (15). However, the Cox-Maze IV procedure has a higher rate of late AF recurrence than the maze-III procedure (16, 17), which remains the main limitation of the Cox-Maze IV procedure. The recurrence of AF after Cox-Maze IV includes early recurrence and late recurrence, and early recurrence is a powerful independent predictor of late recurrence (18). To lower the incidence of late AF recurrence, it is crucial to identify accurate methods to predict the possibility of late recurrence immediately after the Cox-Maze IV procedure.

During the Cox-Maze IV procedure, bipolar ablation forceps and pens are often used to predict the transmural damage of tissue cells by measuring the tissue impedance between both electrodes. However, impedance of the whole tissue measured with bipolar ablation forceps or pens cannot eliminate the possibility of residual connection of focal survival atrial muscle, which can lead to leakage of the lesion line. Some experts suggest that the bacteriological mapping of bidirectional block of key ablation lines after surgical maze ablation of AF should be beneficial (19), which is expected to be the latent end point of surgical maze ablation. In the domain of traditional interventional AF ablation, electrophysiological mapping can not only evaluate the completeness of bidirectional electrical isolation of ablation lines but also induce AF before and after ablation. Hwang ES et al. evaluated the induction of AF by pacing in 89 patients with AF before and after radio-frequency ablation and found that the induction rate of AF decreased from 95.4% before ablation to 56.3% after ablation (20). Theoretically, a relevant mapping technique in interventional cardiology can also be used in the field of surgical maze ablation. Unfortunately, atrial electrophysiological mapping was seldom used in previous surgical maze procedures. Lanters EAH et al performed AF induction in 44 patients with paroxysmal AF who had recovered from sinus rhythm before surgery, yet found no relation between the inducibility of AF before surgery and early postoperative AF (21). Till present, less is known about the relationship between the inducibility of AF after Cox-Maze surgery and late AF recurrence. Therefore, we performed a prospective randomized controlled clinical trial to observe the safety and efficacy of electrophysiological mapping following the Cox-Maze IV procedure (Electrophysio-Maze) and investigate whether a correlation exists between late AF recurrence 6 months after the Cox-Maze procedure with the completeness of electrical isolation of ablation lines in mitral isthmus, left atrial “box,” and tricuspid annulus and with the inducibility of AF immediately after the Cox-Maze IV procedure.

## Materials and methods

This clinical study has been registered on the Chinese clinical trial website (ChiCTR1900023775). This research was funded by the National Key Research and Development Program (2018YFC1311204) and was approved by the ethics committee of the Second Xiangya Hospital of Central South University [2019 ethical review (Scientific Research) No. 054]. Written consent was obtained from all patients before surgery.

### Patient population

Between April 2020 and June 2021, 80 consecutive patients who suffered from aortic valve or mitral valve disease accompanied by persistent AF were randomly enrolled into control group and electrophysiological mapping following the Cox-Maze IV group (Electrophysio-Maze group). The inclusion criteria were as follows: (1) patients of either sex aged between 35 and 70 years; (2) history of valvular disease >3 years; (3) persistent atrial fibrillation (the onset of atrial fibrillation lasts for more than 7 days or requires the use of drugs or electrical cardioversion to convert the heart rhythm); (4) ejection faction >40%; and (5) a clinical diagnosis based on at least one of the following factors: rheumatic mitral stenosis or regurgitation with or without tricuspid regurgitation, rheumatic aortic valve stenosis or regurgitation with or without tricuspid regurgitation, rheumatic both mitral and aortic valve stenosis or regurgitation with or without tricuspid insufficiency, and non-rheumatic mitral, and/or aortic valve stenosis or insufficiency with or without tricuspid insufficiency. The diagnostic criteria for persistent atrial fibrillation were: twice electrocardiograph (ECG) Holter monitoring performed both at the first time more than 7 days ago and at the second time exactly before the surgery confirmed that rhythm all the time is atrial fibrillation without sinus rhythm in any time. While calculating duration of AF, the date of AF onset was defined by the earliest ECG record, which confirmed atrial fibrillation. The exclusion criteria were as follows: (1) previous open-heart surgery and (2) schemed concomitant coronary artery bypass grafting. Previous percutaneous balloon mitral valvuloplasty was not considered an exclusion criterion.

### Surgical procedure

Patients in both groups will undergo mitral or aortic valve replacement or angioplasty, and in the Electrophysio-Maze group, patients will undergo additional concomitant Electrophysiological mapping following the Cox-Maze procedure, where electrophysiology mapping of the completeness of electrical isolation of ablation lines in mitral isthmus, left atrial “box,” and tricuspid annulus will be implemented after Cox-Maze IV. The standard Cox-Maze IV procedure was performed as described previously by Damiano et al. with a combination of bipolar radio-frequency ablation clamp (Medtronic, United States) and bipolar radio-frequency ablation pen MAX3 (AtriCure, United States; 22). Briefly, the patient was in the supine position for the procedure, and then a median thoracotomy was performed, followed by heparinization and establishment of cardiopulmonary bypass. Two incisions were made, one of which is on the right atrium and is parallel to the right atrioventricular sulcus, the other of which is on the left atrium and is longitudinally along the interatrial sulcus. The clamp ablation of the mitral isthmus by bipolar ablation forceps could not reach the mitral annulus, so a “spot ablation” by bipolar radio-frequency pen MAX3 was added to fix the ablation at the corresponding point of the coronary sinus on both epicardial and endocardial surface, and a “sliding ablation” by the bipolar radio-frequency pen was performed on the connecting line between the coronary sinus and mitral annulus on both epicardial and endocardial surface. Each ablation procedure was performed twice. If a thrombus was detected in the left atrium, it was cleared before completing the ablation procedure of the left atrium. After all the ablation routes of the left atrial maze surgery, valvuloplasty, replacement, or intracardiac repair was performed. After closing the left atrium, restoring of sinus rhythm, and opening the ascending aorta, the ablation route of the right atrium was performed.

### Mapping procedure

Mapping of mitral isthmus ablation line: A decapolar electrophysiological catheter was inserted into the coronary sinus, and the proximal end polar (the 10th polar) was just located at the ostium of coronary sinus, with every two adjacent electrodes (e.g., polar 1–2 and polar 3–4) forming a pair of recording electrodes. After sinus rhythm recovered, the proximal electrode pair was paced at a cycle of 400–500 ms (>50 ms shorter than the cycle of the patient’s own heart rate), and the delay from the proximal electrode pair to the distal electrode pair was recorded by a 64-channel physiological function recorder (Jinjiang Electronics, China). If the delay from the proximal electrode pair to the distal electrode pair was >120 ms, the conduction block of ablation line from the proximal atrium to distal atrium was identified as complete; otherwise, it was identified as incomplete. Then, the distal electrode pair was paced at the same cycle, and the delay from the distal electrode pair to the proximal electrode pair was also recorded. If the delay from the distal electrode pair to the proximal electrode pair was >120 ms, the conduction block of ablation line from the distal atrium to proximal atrium was identified as complete; otherwise, it was identified as incomplete.

Mapping of left atrial box ablation lines: the distal electrode pairs of the decapolar electrophysiological catheter were placed in the box, and the proximal polar pairs were placed at the left atrium outside of the box. If there was no atrial potential captured or disturbed by the external sinus rhythm at the electrode pairs in box, the conduction block in the direction from the external left atrium to the box was identified as complete; otherwise, it was identified as incomplete. Then, the distal polar pair in the box was paced in a 400–500 ms cycle (>50 ms shorter than the cycle of the patient’s own sinus rate). If the left atria outside of the box was not captured or disturbed by pacing, the conduction block in the direction from the box to the external left atrium was identified as complete; otherwise, it was identified as incomplete.

Mapping of the tricuspid annulus ablation line: A decapolar electrophysiological catheter vertically crossed the tricuspid annulus ablation line, and the overall electrode array was distributed on both sides of the ablation line as symmetrically as possible, with the tip pointing to the free side wall of the right atrium (pointing to the right side of the patient’s body) and the most proximal electrode pair close to the anterior atrial sulcus. A proximal electrode pair was paced at a cycle of 400–500 ms (>50 ms shorter than the cycle of the patient’s own heart rate), and the delay from the proximal electrode pair to the distal electrode pair was recorded. If the delay was more than 120 ms, the conduction block of ablation line from the proximal atrium to distal atrium was identified as complete; otherwise, it was identified as incomplete. Then, a distal electrode pair was paced at a similar cycle, and if the delay from the distal electrode pair to the proximal electrode pair was >120 ms, the conduction block of ablation line from the distal atrium to proximal atrium was identified as complete; otherwise, it was identified as incomplete.

### Possible complementary ablation

If the electrophysiological mapping immediately after maze operation indicates that the ablation line of tricuspid annulus in incomplete, whether to conduct complementary ablation will be independently decided by operator according to the stability of the patient’s clinical condition. The supplementary ablation method of tricuspid annulus is to use a bipolar radio-frequency pen to perform “sliding ablation” twice on the previous tricuspid annulus ablation line on the epicardial surface. Then, the mapping of tricuspid annulus ablation line was performed again. If electrophysiological mapping indicates that the bidirectional isolation of mitral isthmus or box ablation lines is incomplete, so as to avoid additional risks of supplementary ablation, which needs to flip the heart, complementary ablation was forbidden.

### Atrial fibrillation induction procedures before and after Cox-Maze IV

Atrial fibrillation induction before Cox-Maze IV was performed immediately after cannulization and before cardiopulmonary bypass. The tail line of bipolar radio-frequency ablation pen MAX3 was connected to the 64-channel physiological function recorder, with two tip electrodes placed in the high right atrium. The tip electrode pair was stimulated successively at a rate of 400, 300, 250, 230, 210, 190, 170, and 150 ms cycles, for 10 s in each cycle. If AF was induced and sustained for more 30 s, atrial fibrillation induction was identified as positive, and then electrical cardioversion was performed.

After the cardiopulmonary bypass was terminated and before the cannulization was withdrawn, AF induction procedures after Cox-Maze IV were performed again. The induction procedure was the same as that carried out before Cox-Maze IV.

### Postoperative management

All patients in two groups received amiodarone to prevent early postoperative recurrence of AF for 3 months. Electrical cardioversion was not a choice regardless of possible in-hospital AF recurrence if the hemodynamics was stable. If there was degree 3 atrioventricular conduction block that did not recover 7–10 days after surgery, a rate-responsive atrioventricular pacemaker will be implanted.

### Follow-up

All patients in two groups were continuously followed-up and underwent a 24 h ECG Holter monitoring after 6 months, where the late AF reccurrence is defined as AF or atrial flatter lasting for more than 30 s. AF onset within 3 months after surgery, which is known as the postsurgical blanking period, will not be classified into late AF reccurrence. Beside the schemed ECG Holter monitoring after 6 months, if a patient reported persistent symptoms such as palpitation between 3 and 6 months, he/she will be recommended with an immediate ECG examination at the nearest hospital. Under this condition, if any AF onset lasting for more than 30 s was confirmed by ECG, the diagnostic of late AF recurrence is also established. Both follow-up and medical treatment were performed by doctors independent of main researchers.

### Statistics

All statistical analyses were performed using the SPSS software (version 21.0, IBM Corporation, United States). For quantitative variables, normality was assessed using the Shapiro–Wilk test, and mean ± SD was used to summarize normally distributed data. Normal distributions were achieved by logarithmic transformation, if necessary. While comparing continuous variables between two groups, analysis of variance was used to test the homogeneity of the variances of the two groups. If the variances are consistent, Student’s *t*-test was adopted; otherwise, Dunnett’s *t*-test should be an alternative selection. For the correlation between qualitative variables, the chi-square test was used, and the corresponding odds ratio was analyzed. All tests were two-tailed, and the significance level was set as 0.05, taking 95% confidence intervals.

## Results

### Demographics

Totally, 42 patients were enrolled into the Electrophysio-Maze group, and 38 patients were enrolled into control group. Between two groups, there were no difference in sex, age, duration of AF, long-standing AF percent, Euroscore II score, hypertension, coronary heart disease, preoperative cardiovascular accident, preoperative TIA, left atrial diameter, right atrial diameter, left ventricular diastolic diameter, right ventricular diastolic diameter, ejection fraction, and the use of antiarrhythmic drugs (Table 1).

**TABLE 1:** Baseline characteristics of two groups.

Items	Electrophysio-Maze group	Control group	Statistical value	*P*-value
Sex,% of males	28.6% (12/42)	47.4%(18/38)	3.01 (χ^2^)	0.083
Age, years	53.0 ± 8.5	56.0 ± 8.6	–1.585 (*t*)	0.117
Duration of AF, months	43.7 ± 47.0	46.8 ± 49.7	–0.282 (*t*)	0.779
Long-standing AF,%	21.4 (9/42)	21.1% (8/38)	0.002 (χ^2^)	0.967
Euroscore II	2.3 ± 1.2	2.2 ± 1.2	0.096 (*t*)	0.924
Hypertenton	4.76% (2/42)	13.16% (5/38)	1.761	0.248
Coronary heart disease	4.76% (2/42)	7.89% (3/38)	0.334	0.664
Cardiovascular accident,%	11.9% (5/42)	10.5% (4/38)	0.038 (χ^2^)	0.846
Preoperative TIA,%	9.5% (4/42)	2.6% (1/38)	1.617 (χ^2^)	0.203
LA, mm	50.85 ± 8.32	51.11 ± 12.68	–0.104	0.917
RA, mm	36.33 ± 3.47	37.47 ± 5.26	–1.131	0.262
LV, mm	49.40 ± 8.09	48.18 ± 7.36	0.703	0.484
RV, mm	35.12 ± 2.78	36.89 ± 4.87	–1.420	0.161
EF,%	60.71 ± 8.92	61.13 ± 12.50	–0.173	0.863
Antiarrhythmic drugs,%	100 (42/42)	100 (38/38)	0	1

### Comparison of perioperative characteristics between two groups

There were no statistical difference in type of valve operation (percent of mitral valve and aorta valve), left atrial appendage thrombosis and temporary pacing, intraoperative blood loss, and postoperative total drainage volume between two groups. No patient was implanted with a permanent pacemaker, and no patient died or underwent stroke during hospitalization in both groups. Compared with control group, patients in the Electrophysio-Maze group underwent longer bypass time and cross-clamp time. In the Electrophysio-Maze group, a patient underwent rehospitalization due to chest wound infection and recovered after debridement, but there are no statistical differences in rehospitalization between two groups. Interestingly, Electrophysio-Maze can significantly shorten the length of hospitalization stay (Table 2).

**TABLE 2:** Comparison of perioperative characteristics between two groups.

Items	Electrophysio-Maze group	Control group	Statistical value	*P*-value
Mitral valve surgery,%	100 (42/42)	94.74 (36/38)	2.267 (χ^2^)	0.132
Aorta valve surgery,%	35.71 (15/42)	42.11 (16/38)	0.002 (χ^2^)	0.558
Left atrial appendage thrombosis,%	4.8 (2/42)	13.2 (5/38)	1.761	0.184
Bypass time	129.33 ± 31.63	96.37 ± 21.55	4.591 (*t*)	<0.001
Cross-clamp time	81.98 ± 19.98	60.84 ± 25.72	4.126 (*t*)	<0.001
Intraoperative blood loss	282.1 ± 34.6	269.5 ± 37.3	1.575 (*t*)	0.119
Postoperative total drainage	474.5 ± 99.1	450.0 ± 125.1	0.976 (*t*)	0.332
Temporary pacing,%	2.38 (1/42)	5.26 (2/38)	0.459 (χ^2^)	0.498
Length of stay, day	10.9 ± 3.0	13.5 ± 2.9	–3.912 (*t*)	<0.001
Re-hospitalization,%	2.38 (1/42)	0 (0/38)	0.916 (χ^2^)	0.338
Mortality,%	0 (0/42)	0 (0/38)	0 (χ^2^)	1
Stroke,%	0 (0/42)	0 (0/38)	0 (χ^2^)	1

### Results of electrophysiological mapping and complementary ablation in Electrophysio-Maze group

**FIGURE 1:**
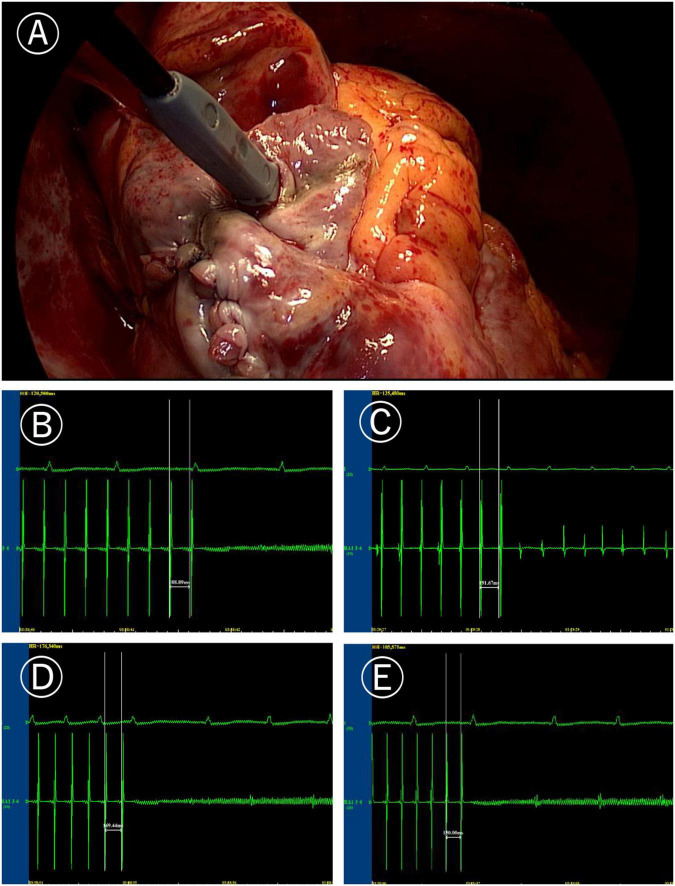
Induction of atrial fibrillation (AF). **(A)** Image of programmed stimulation at high right atrial for induction of atrial fibrillation (post surgery). **(B)** AF induced by the 190 ms cycle stimulation before Cox-Maze IV. **(C)** After Cox-Maze IV, the 190 ms cycle stimulation failed to induce AF again. **(D)** After Cox-Maze IV, the 170 ms cycle stimulation could not induce AF; and **(E)** After Cox-Maze IV, the 150 ms cycle stimulation could not induce AF.

**FIGURE 2:**
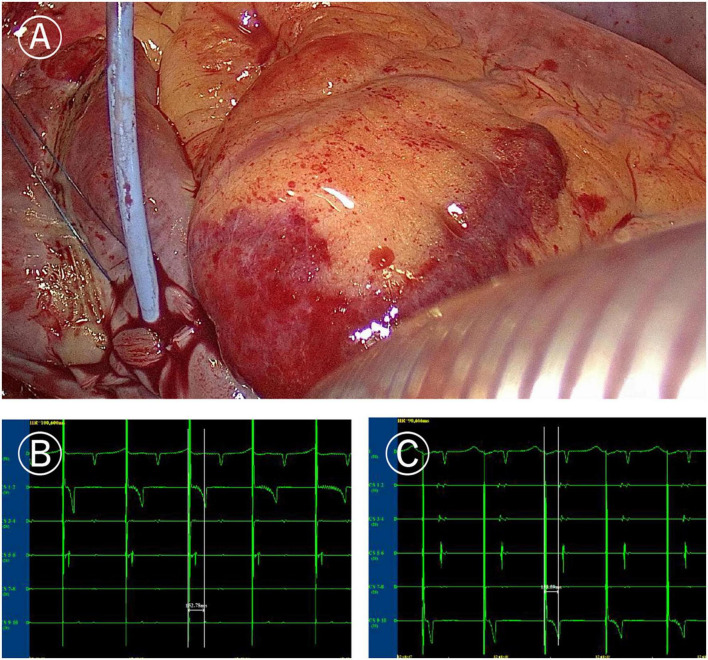
Ablation line mapping of mitral isthmus. **(A)** Coronary sinus catheter mapping of mitral isthmus ablation line. **(B)** Distal (CS1-2) delay during proximal (CS9-10) pacing of coronary sinus catheter (more than 120 ms). **(C)** Proximal delay during distal pacing of coronary sinus catheter (more than 120 ms).

**FIGURE 3:**
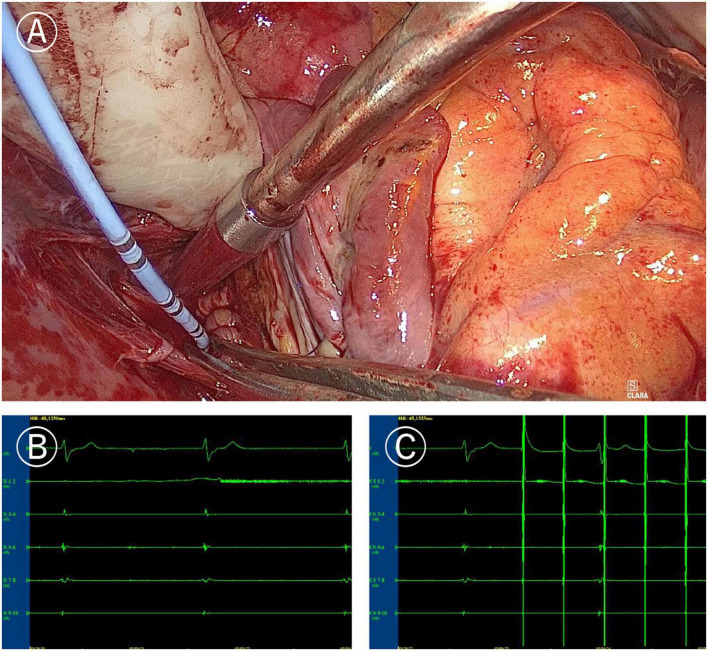
Mapping of left atrial “box” lesion. **(A)** Coronary sinus catheter mapping of left atrial “box” lesion. **(B)** Inner-box polar could not sense the outer potential under sinus rhythm. **(C)** Pacing of inner-box polar could not capture or disturb the sinus rhythm of the outer atrium.

**FIGURE 4:**
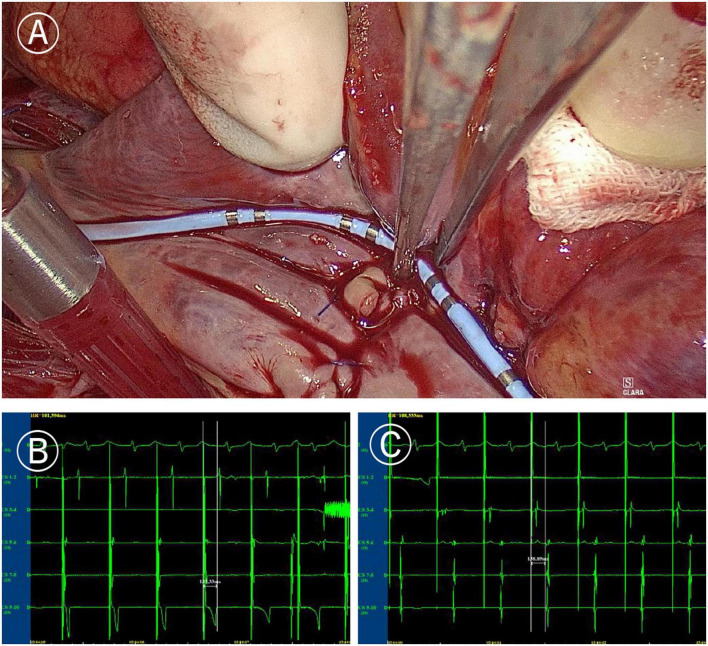
Mapping of tricuspid valve annular ablation line. **(A)** Coronary sinus catheter mapping of tricuspid valve annular ablation line. **(B)** Distal polar in free side wall of the right atrium (CS1-2) delay during proximal polar near the anterior atrial sulcus (CS9-10) pacing of coronary sinus catheter (more than 120 ms). **(C)** Proximal delay during distal pacing of coronary sinus catheter (more than 120 ms).

In the Electrophysio-Maze group, all patients underwent AF induction before and after the Cox-Maze IV procedure (Figure 1). The induction was performed with cannulization but without cardiopulmonary bypass, making the conditions consistent as much as possible. AF was induced in 88.1% (37/42) of all patients before Cox-Maze IV and was induced in 14.3% (6/42) of all patients after the Cox-Maze IV procedure. The bidirectional electrical isolation of the mitral annular isthmus ablation line (Figure 2), “box” ablation lines (Figure 3), and tricuspid annular ablation line (Figure 4) was successfully mapped in each patient. The rate of incomplete bidirectional electrical isolation of “box” ablation lines immediately after Cox-Maze IV was zero (the rate of complete bidirectional conduction block of “box” ablation lines immediately after Cox-Maze IV was 100%), and the rate of incomplete bidirectional electrical isolation of mitral isthmus ablation lines or tricuspid annulus ablation lines was 23.8% (10/42), wherein the rate of incomplete bidirectional electrical isolation of both mitral isthmus and tricuspid annulus ablation line was 7.14% (3/42), the rate of incomplete bi-directional electrical isolation of single mitral isthmus ablation line was 7.14% (3/42), and the rate of incomplete bidirectional electrical isolation of single tricuspid annulus ablation line was 9.52% (4/42). In two cases of incomplete bidirectional electrical isolation in single tricuspid annulus ablation line, complementary ablation was performed, and then electrophysiological mapping was performed again to ensure that the subsequent electrophysiological mapping confirmed the final complete bidirectional electrical isolation. The final incomplete bidirectional electrical isolation rate of mitral isthmus or tricuspid annulus ablation line was 19.0% (8/42). The incomplete bidirectional electrical isolation rate of ablation lines was correlated with AF inducibility immediately after Cox-Maze IV procedure but not with AF inducibility before Cox-Maze IV (Table 3).

**TABLE 3:** Correlation between incomplete conduction blocking of ablation lines with atrial fibrillation (AF) inducibility before and after Cox-Maze IV in the Electrophysio-Maze group.

Items		Complete immediate blocking	Incomplete immediate blocking	Chi-square	*P*-value
Induction before Cox-Maze IV	No	5	0	1.335	0.564
	Yes	29	8		
Induction after Cox-Maze IV	No	34	2	29.750	<0.001
	Yes	0	6		

### Comparison of follow-up results after 6 months between two groups

No patient died or suffered stroke during follow-up in both groups. All patients were prescribed with amiodarone for 3 months and underwent 24 h ECG Holter after 6 months. Compared with patients in the control group, patients in the Electrophysio-Maze group experienced more obvious reduction of the inner diameter of left atrium, right atrium, left ventricle, and right ventricle, a greater increase of left ventricular ejection fraction, and a higher relief from AF at 6 months (88.1 vs. 13.2%, *P* < 0.001, refer to Table 4).

**TABLE 4:** Comparison of follow-up results after 6 months between two groups.

Items	Electrophysio-Maze group	Control group	Statistical value	*P*-value
Mortality,%	0 (0/42)	0 (0/38)	0 (χ^2^)	1
Stroke,%	0 (0/42)	0 (0/38)	0 (χ^2^)	1
LA, mm	38.95 ± 5.26	42.92 ± 6.32	–3.063 (*t*)	0.003
RA, mm	30.26 ± 3.21	34.50 ± 4.21	–5.180 (*t*)	<0.001
LV, mm	41.86 ± 4.76	44.29 ± 5.45	–2.132 (*t*)	0.036
RV, mm	23.86 ± 3.32	33.42 ± 3.28	–12.937 (*t*)	<0.001
EF,%	71.10 ± 4.54	67.29 ± 9.25	2.299 (*t*)	0.026
Free of AF,%	88.1 (37/42)	13.2 (5/38)	44.925 (χ^2^)	<0.001

### Correlation between atrial fibrillation recurrence after 6 months and results of electrophysiological mapping in operation

There was a significant correlation between AF recurrence at 6 months both with incomplete block of ablation lines and with AF inducibility immediately after Cox-Maze IV during the operation. Additionally, AF recurrence at 6 months was correlated with a large left atrium (Table 5).

**TABLE 5:** Correlation between AF recurrence with final incomplete blocking, AF inducibility, and large left atrial in the Electrophysio-Maze group.

Items		Free of AF	AF recurrence	Chi-square	*P*-value
Final incomplete blocking	No	33	1	13.675	0.003
	Yes	4	4		
AF induction after MAZE	No	34	2	9.686	0.015
	Yes	3	3		
Large left atrial (>80 mm)	No	32	2	6.173	0.040
	Yes	5	3		

## Discussion

In this study, we reported outcomes of a randomized controlled clinical trial of electrophysiological mapping following the Cox-Maze IV procedure and found that compared with patients in control group, those in the Electrophysio-Maze group experienced shorter hospital stay, better cardiac remodeling changes, and higher relief from AF during follow-up period of 6 months, thus showing similar advantages of Cox-Maze IV as reported in previous studies (23, 24). Most importantly, we found that the rate of bidirectional electrical isolation of “box” ablation lines immediately after Cox-Maze IV were 100%. As the “box” area is the necessary “path” for the four pulmonary vein to connect the main body of left atria, theoretically, all the latent intracavitary pulmonary vein triggers were abolished, which substitutes the basis of the prominent efficacy of maze surgery. However, there was incomplete bidirectional electrical isolation in the mitral isthmus and tricuspid annulus ablation lines in part patients. Interestingly, in two cases of incomplete bidirectional electrical isolation of single tricuspid annulus ablation line, complementary ablation was successful. Both incomplete bidirectional electrical isolation of ablation lines and AF induction immediately after the maze procedure were found to be significantly correlated with late AF recurrence after 6 months.

Incomplete bidirectional isolation of the ablation line has been reported to form the anatomical basis of late AF recurrence in catheter-based AF ablation (25), yet there was no similar concomitant electrophysiological mapping study in domain of open surgery for Cox-Maze IV. Some hybrid ablation studies also reported the result of electrophysiological mapping in pulmonary vein isolation (PVI). Two studies on thoracoscopic encircling catheter-based ablation (26, 27) and a thoracoscopic bipolar radiofrequency clamp ablation (28) all reported 100% leakage in “box” ablation line immediately after ablation, which partly explains why bidirectional electrical isolation test of left atrial “box” ablation lines is not routinely carried out in catheter or thoracoscopic ablation in the real world. In a hybrid maze (minimally invasive surgical ablation combining second-staged catheter mapping and provisional ablation) research on ablation of isolated AF, there was a gap in 23% (5/22) boxes ablation lines and 100% (3/3) mitral isthmus lines (29). In another report on surgical thoracoscopic left atrial box radiofrequency ablation by bipolar clamp combining bipolar linear radiofrequency pen in persistent and long-standing persistent AF patients, gaps in PVI lesions existed in 87% right PV, 77% left PV, 67% roof lines, and 40% inferior lines (30). In this study, the completeness of the surgical Cox-Maze IV procedure in left atrial box ablation lines, mitral isthmus ablation line, and tricuspid valvular ablation line seems much better than interventional catheter ablation and thoracoscopic clamp ablation studies mentioned above. We postulated that the high rate of complete bidirectional electrical isolation in pulmonary vein vestibules insulation and box ablation might result not only from the standard protocol of the Cox-Maze IV procedure in this study but also from the operational stability of a fixed operator who completed the largest number of maze procedures in China.

The induction of AF can be divided into drug-based and electrophysiological-stimulation (pacing) methods, and the latter is used more commonly. In a hybrid AF research, 15 patients with persistent or long-standing persistent AF who failed at least one catheter ablation and one antiarrhythmic drug intervention underwent surgical ablation, followed by AF induction after several days; the AF could be induced by atrial rapid pacing in nearly half of all enrolled patients after surgical ablation (31). Hwang ES evaluated the AF induction rate by pacing before and after catheter radiofrequency ablation in 89 patients with AF and found that ablation of AF reduced the induction rate of AF from 95.4% before operation to 56.3% after ablation (20, 32). Santangeli et al. proposed that the standardized induction scheme of AF should include electrical stimulation-induced AF and infusion of high-dose isoproterenol (33). Theoretically, the combination of drugs and electrical stimulation can improve the induction rate of AF, but it will inevitably increase the false positive rate. Hence, in this study, we only adopted the pacing method in AF induction. Peter Leong-Sit et al conducted a prospective prognostic research of AF induction rate after PVI AF ablation in 144 patients (34), and found that the inducible AF after AF ablation occurred in 52 cases (36.1%). The inducibility of AF after ablation in the above-mentioned studies was much higher than in this study (about 14%). The prognostic value of AF inducibility on the long-term prognosis has been researched in catheter-based ablation by Kosiuk et al. (35), wherein the AF inducibility had no effect on early recurrence but could affect late recurrence, which is consistent with the results of this study.

Generally speaking, almost all previous research in the open Cox-Maze surgery domain (except staged hybrid surgery of surgical and subsequent electrophysiological mapping and complementary catheter ablation) focused solely on ablation strategy based on anatomy, and the importance of electrophysiological mapping was ignored more or less. So, the feasibility and effectiveness of electrophysiological assistance during open Cox-MAZE IV are unclear. This is the first study to demonstrate that electrophysiological mapping can guide supplementary ablation and increase ablation success rate, and that AF induction after the open Cox-MAZE IV procedure can predict the recurrence of atrial fibrillation 6 months after the standard Cox-MAZE IV procedure. Findings in this study bring new strategies not only in understanding the late AF recurrence after Cox-Maze IV but also in screening high-risk populations of late AF recurrence and developing possible preventative measures to lower the incidence of late AF recurrence after Cox-Maze IV. To reduce the risk of late AF recurrence, future studies should focus on lowering the leakage rate in valvular annulus ablation lines and the AF inducibility immediately after ablation. Furthermore, this study can provide a latent protocol of electrophysiological mapping, AF induction, and supplementary ablation for future open Cox-Maze IV studies. It should be pointed out, concerning the complexity of completing electrophysiological mapping, Cox Cox-Maze IV procedure and valvular surgery in an operation, a fixed skilled operator who has passed the learning curve is needed to improve the success rate of AF ablation and avoid unexpected complications associated with concomitant valve surgery. For example, according to the results of a recent randomized trial, the frequency of permanent pacemaker implantation was 7.7% in patients undergoing mitral valve surgery alone, 16.1% in patients undergoing mitral valve surgery plus PVI, and 25% in patients undergoing mitral valve surgery plus biatrial ablation (36), indicating that maze procedure is associated with an increased risk of sinus node dysfunction and permanent pacemaker implantation (37). However, there was no complication of high atrioventricular conduction block requiring permanent pacemaker implantation in this study, which once again proved the importance of sophisticated surgical skills while carrying out the Cox-Maze IV procedure concomitant with valvular surgery.

This study has some limitations. First, this study only mainly focused on the bidirectional electrical isolation of mitral annular isthmus ablation line, left atrial “box” lines, and tricuspid annulus line, without involving other lines such as superior vena cava ablation line, inferior vena cava ablation line, and left atrial appendage ablation line. One of the main reasons is that there is no normal reference value to judge the complete conduction block in such ablation lines at present. Second, although testing for inducibility of AF was performed under some conditions (e.g., both are off pump) before and after ablation, there are residual metabolic changes after extracorporeal circulation that can affect inducibility. Yet, these metabolic changes often increase the risks of atrial fibrillation called postoperative atrial fibrillation (38), which might partially reduce the efficacy of maze surgery, so it would not hinder the rationality of the conclusion that Cox-Maze IV is effective in reducing the rate of atrial fibrillation inducibility. Third, before the surgical procedure, a mitral valvular disease could be related to atrium enlargement and early onset of asymptomatic AF, where the duration of AF in some patients might be underestimated. Fortunately, this impact was exerted on both group, so it was a random error rather than a systemic error, whose influence was controllable in the controlled study. Fourth, secondary causes of AF should be considered and were not investigated. These forms could not respond to ablation approach including an early onset of unknown atrial fibrillation in channelopathies like Brugada (39) and long QT sindrome (40), hypertension, or hyperthyroidism (41), and also lifestyle factors such a regular practice of endurance sport (42). Fifth, although the rate of complete bi-directional electrical isolation of “box” ablation lines was found to be 100% immediately after Cox-Maze IV, yet whether there is pulmonary vein reconnection after 6 months is unknown. As an important trigger of AF recurrence, the latent impact of pulmonary vein reconnection should be considered while analyzing the reason for AF recurrence after 6 months. Due to the difficulty in persuading patients to undergo a postoperative electrophysiological examination, this study did not perform intracardiac electrophysiological examination for all patients suffering late AF recurrent.

## Conclusion

Electrophysiological mapping following the Cox-Maze procedure is safety and effective in shortening hospital stay, leading to better cardiac remodeling changes and improving relief from atrial fibrillation during follow-up period of 6 months. Electrophysiological mapping in the Cox-Maze procedure can find out the non-transmural annulus ablation line by assessing the completeness of bidirection electrical isolation of ablation lines, guide supplementary ablation, and predict atrial fibrillation recurrence after 6 months.

## Data Availability Statement

The raw data supporting the conclusions of this article will be made available by the authors, without undue reservation.

## Ethics statement

The studies involving human participants were reviewed and approved by the Ethics Committee of the Second Xiangya Hospital of Central South University. The patients/participants provided their written informed consent to participate in this study.

## Author contributions

LL: conceptualization and project administration. ZS, HT, and YS: data curation. ZS: formal analysis and writing–original draft. CF, LS, and LL: investigation. HZ: methodology. ZJ: resources. CF and LL: supervision and writing–review and editing. All authors contributed to the article and approved the submitted version.

## Funding

This study was supported by the National Key Research and Development Program (No. 2018YFC1311204 to LL).

## Conflict of Interest

The authors declare that the research was conducted in the absence of any commercial or financial relationships that could be construed as a potential conflict of interest.

## Publisher’s Note

All claims expressed in this article are solely those of the authors and do not necessarily represent those of their affiliated organizations, or those of the publisher, the editors and the reviewers. Any product that may be evaluated in this article, or claim that may be made by its manufacturer, is not guaranteed or endorsed by the publisher.
